# The role and function of lncRNA in ageing-associated liver diseases

**DOI:** 10.1080/15476286.2024.2440678

**Published:** 2024-12-19

**Authors:** Peyman Kheirandish Zarandi, Mohsen Ghiasi, Mohammad Heiat

**Affiliations:** a Department of Biology, Science and Research Branch, Islamic Azad University, Tehran, Iran; b Cancer Biology Signaling Pathway Interest Group (CBSPIG), Universal Scientific Education and Research Network (USERN), Tehran, Iran; c Rajaie Cardiovascular Medical and Research Center, Iran University of Medical Sciences, Tehran, Iran; d Baqiyatallah Research Center for Gastroenterology and Liver Diseases (BRCGL), Baqiyatallah University of Medical Sciences, Tehran, Iran

**Keywords:** Liver diseases, ncRNAs, lncRNAs, ageing, liver ageing

## Abstract

Liver diseases are a significant global health issue, characterized by elevated levels of disorder and death. The substantial impact of ageing on liver diseases and their prognosis is evident. Multiple processes are involved in the ageing process, which ultimately leads to functional deterioration of this organ. The process of liver ageing not only renders the liver more susceptible to diseases but also compromises the integrity of other organs due to the liver’s critical function in metabolism regulation. A growing body of research suggests that long non-coding RNAs (lncRNAs) play a significant role in the majority of pathophysiological pathways. They regulate gene expression through a variety of interactions with microRNAs (miRNAs), messenger RNAs (mRNAs), DNA, or proteins. LncRNAs exert a major influence on the progression of age-related liver diseases through the regulation of cell proliferation, necrosis, apoptosis, senescence, and metabolic reprogramming. A concise overview of the current understanding of lncRNAs and their potential impact on the development of age-related liver diseases will be provided in this mini-review.

## 1. Introduction

Liver disease is responsible for more than two million fatalities each year, including deaths caused by fibrosis, cirrhosis, viral hepatitis, and liver cancer [1,2]. Increasing evidence suggests that the elderly are at a greater risk of developing liver diseases [3]. A worldwide elderly population is experiencing an increase in the prevalence of chronic liver disease; this will inevitably lead to a greater number of fatalities among this age group. These individuals thus necessitate increased levels of healthcare [4].

**Figure 1.**
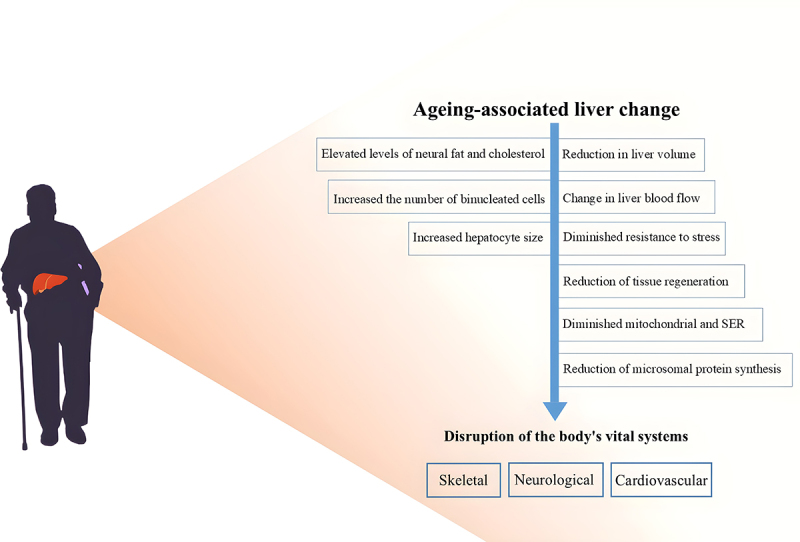
The impact of ageing on liver function and its role in disorders affecting the skeletal, neurological, and cardiovascular systems.

The liver, as one of the largest organs of the body, plays a very important role. Liver-derived substances, particularly hepatokines, modulate the energy homoeostasis and physiological processes of bones, the neurological system, the heart, and adipose tissue. Consequently, ageing of the liver may significantly contribute to the development of diseases in other organs [5]. The ageing of this very important organ is associated with morphological and functional changes such as volume reduction, reduced tissue regeneration ability, and significant changes in liver blood flow [6]. Diagnostic examinations using multidetector computed tomography have clearly shown that liver volume constantly diminishes with ageing [7]. According to recent research, decreased blood flow indicators in the main portal vein over the age of 60 are primarily responsible for the reduction in liver volume and weight [8]. Furthermore, the decrease in both the number and size of liver cells also affects this process [9]. Actually, the number and volume of these cells increase gradually until maturity; however, they thereafter decline with ageing [10]. Ageing directly affects different liver cells, leading to numerous phenotypic changes [11]. A further consequence of ageing is a diminished resistance to stress, which renders the liver more susceptible to toxic compounds [3]. As people age, their liver’s neural fat and cholesterol contents increase, which may cause organ-specific toxic responses and harm liver function. Furthermore, liver ageing may reduce mitochondrial number, function, and smooth endoplasmic reticulum area. As a result of these implications, microsomal protein synthesis is diminished [12]. The outcomes of liver ageing are illustrated in Figure 1.

Numerous non-genetic factors, including environmental influences, lifestyles, alcohol consumption, and exposure to toxic substances, heighten the elderly’s vulnerability to chronic liver inflammation [13–15]. Elderly people consuming an inappropriate diet, characterized by high levels of refined carbohydrates, particularly fructose, and saturated fats, while lacking in fibres, polyunsaturated fats, and vitamins, are a primary contributing cause to non-alcoholic fatty liver disease (NAFLD) [16]. Also, studies have shown a positive correlation between age and NAFLD in older people [17]. The most prevalent causes of mortality associated with chronic liver disease are NAFLD and its progressive form, non-alcoholic steatohepatitis (NASH) [18]. The incidence of NASH is anticipated to rise due to the epidemics of obesity, metabolic syndrome, type 2 diabetes mellitus, insulin resistance, hypertension, and cardiovascular illnesses [18,19]. Numerous investigations have demonstrated that NASH and its associated complications, including cirrhosis, hepatocellular carcinoma, and progressive fibrosis, have a disproportionate impact on elderly patients [18,20–22]. The biopsies from elderly NASH patients show shorter telomeres, elevated p21 levels, and an enhanced DNA damage response with γH2AX. Research has shown that a higher level of p21 expression in hepatocytes is associated with the illness stage, type 2 diabetes, and negative consequences [23,24]. As previously indicated, the progression of liver diseases, including chronic hepatitis in the elderly, can be accelerated by alcohol consumption, which is a non-genetic factor. The most prevalent and deadly side effect of long-term heavy drinking is alcohol-associated liver disease (ALD). ALD includes hepatic steatosis, cirrhosis, and liver cancer [25]. Elderly patients are more susceptible to developing alcoholic liver cirrhosis than their younger counterparts, as ALD is more prevalent in these populations [26]. The liver’s detoxification capacity is significantly reduced in elderly individuals with one or more co-morbidities, which not only renders them predisposed to alcohol toxicity and carcinogenesis but also results in a poor prognosis [27]. In an experiment on old mice and ethanol-fed mice, neutrophilic SIRT1 expression was significantly downregulated in both groups. Interestingly, the downregulation of neutrophilic SIRT1, associated with age and alcohol, results in diminished expression of the anti-inflammatory and anti-fibrotic microRNA-223 (miR-223), hence increasing vulnerability to ALD. Moreover, findings indicated that SIRT1 downregulation in neutrophils, induced by ageing or alcohol, promoted the acetylation of CCAAT/enhancer-binding protein α (C/EBPα), a transcription factor that regulates miR-223 production [28].

Ageing is a very complex process involving multiple cellular and molecular pathways. Various stresses aggravate the ageing process. Processes such as oxidative stress and genotoxic stress have been identified in age-related diseases [29,30]. Cellular senescence can be defined as a biological, intrinsic and time-dependent decay that is associated with the shortening of the telomere length of chromosomes, modifications in epigenetics, disrupted protein homoeostasis, dysfunction of mitochondria, metabolism, immunosenescence and inflamm-ageing [3,31,32]. The ageing process is associated with the condition of immunosenescence, which is characterized by a decrease in the capacity to respond to pathogens. This decline affects both innate and adaptive immune pathways [33]. Inflamm-ageing is a disorder that is characteristic of old age and is characterized as a persistent, sterile, systemic inflammatory disease. In the exhaustive examination of age-related immunological decline, it is possible to recognize that immunosenescence and inflamm-ageing, despite their apparent contradictions, are two distinct aspects of the same phenomenon [34]. Lately, there has been a growing focus on the significant and crucial involvement of numerous non-coding RNAs (ncRNAs) in all aspects of the cellular ageing process, including transcriptional, post-transcriptional, translational, and post-translational levels [35,36].

Research has shown that a significant portion of human DNA is transcribed into non-protein-coding RNA, which has been conclusively proven to play a complicated role in physiological processes. Important biological roles of ncRNAs, including long non-coding RNAs (lncRNAs), microRNAs (miRNAs) and circular RNAs (circRNAs), have been shown in several disorders [37–40], with a particular emphasis on liver disease [41–44]. Previous research has conclusively demonstrated that numerous ncRNAs exhibit differential expression levels between the ageing and youthful states of the liver in mice, pigs and humans [12,45,46]. LncRNAs are RNA transcripts that are longer than 200 nucleotides and do not encode proteins. LncRNAs are very important functional units whose intracellular location is crucial for their function [47]. Studies show that lncRNAs can play a role in processes such as cell proliferation, suppression of apoptosis, promotion of angiogenesis, migration, and metastasis. In addition, lncRNAs play a very important role in metabolic reprogramming and senescence [48–50]. In this mini-review, we intend to discuss the role of lncRNAs in the ageing process of the liver and provide a new perspective on the important role of lncRNAs in the ageing process of liver cells.

## 2. Exploring the functions and mechanisms of lncRNAs

The classification, functions, and mechanisms of lncRNAs in biological organisms are highly intricate. Categorized into six groups based on their genomic position in relation to protein-coding genes, lncRNAs include sense/antisense exonic, sense/antisense intronic, intergenic, and bidirectional types. These molecules play vital roles in various diseases and biological processes like cancer, differentiation, cell death, growth, and survival. Through diverse mechanisms, lncRNAs can influence biological pathways and cellular functions, such as targeting transcription factors, hindering neighbouring gene transcription, guiding methylation processes, and initiating changes in chromatin structure [51–53].

## 3. The role of lncRNAs in liver regeneration

The process of liver regeneration involves the growth of liver tissue following damage or loss, characterized by compensatory hyperplasia rather than true regeneration. Hepatocyte proliferation primarily drives this process [54]. Liver regeneration consists of three separate stages: priming, proliferation, and restoration, which are all subject to regulation by multiple factors [55]. The study on mouse model liver cells revealed that lncRNA MALAT1 regulates liver cell proliferation during liver regeneration. This research revealed an upregulation of MALAT1 during liver regeneration [56]. Haijing B et al. used high-throughput sequencing techniques to identify lncRNAs selectively expressed during rat liver regeneration. In this study, 10 key lncRNAs and 10 key mRNAs were determined. NONRATT003557.2, NONRATT005357.2, NONRATT003292.2, NONRATT001466.2, NONRATT003289.2, NON-RATT001047.2, NONRATT005180.2, NONRATT004419.2, NONRATT005336.2, and ONRATT005335.2 are among the lncRNAs that have been recognized [54]. Liver regeneration following liver injury or partial hepatectomy is a meticulous and well-organized biological process that involves the induction of synchronized cell proliferation [57]. The critical functions of lncRNAs in modulating liver regeneration, especially after partial hepatectomy, have been extensively researched [55,58]. A mouse microarray study revealed that liver regeneration alters 400 lncRNAs, significantly up-regulating lncRNA-PHx2. The depletion of lncRNA-PHx2 leads to a temporary surge in hepatocyte proliferation and accelerates liver regeneration, a phenomenon that correlates with the up-regulation of genes involved in cell proliferation. LncRNA-PHx2 increased expression at 60 hours after partial hepatectomy, indicating a critical phase of hepatocyte proliferation [59]. Physical damage to the liver, such as that resulting from partial hepatectomy, correlates with heightened histological injury and necrosis. This injury triggers proliferation in the remaining unharmed hepatocytes to compensate for and replace the destroyed liver tissue. Hepatocytes are in an active state of proliferation during liver regeneration, which is associated with an increase in cellular protein synthesis [60]. The lncRNA-Hand2 is a branching lncRNA found near the coding gene Hand2, showcasing the complex relationship between lncRNAs and liver regeneration. Following hepatectomy in mouse liver cells, lncRNA-Hand2 is significantly upregulated and plays a crucial role in regulating NK homeobox 1–2 (Nkx1–2) levels by recruiting the Ino80 remodelling complex. This activation of c-Met signalling pathways is essential for liver regeneration [61].

## 4. LncRNA in liver ageing

Previous studies have shown that lncRNAs have multiple functions during ageing in various tissues and organs, such as the liver [62]. LncRNAs have a crucial role in the aetiology and progression of NAFLD; however, the precise molecular mechanism remains unidentified. In a study conducted in 2021, it was found that lncRNA MAYA causes ageing of liver cells through inhibiting Yes‐associated protein (YAP) in NAFLD. They utilized the golden hamster as a model for NAFLD due to the resemblance between lipid distribution and metabolism in humans [63]. Uncontrolled NAFLD may lead to liver damage, severe fibrosis, cirrhosis, and hepatocellular carcinoma [63]. To gain insight into the transcriptional mechanisms underlying human liver ageing, one study utilized the pig as the desirable animal model. Screening lncRNAs identified in porcine liver during ageing was performed to identify age-related lncRNA expression profiles. The screening results identified nineteen lncRNAs that exhibited differential expression in the aged liver of porcine. The findings indicated that the target genes of lncRNAs that were up-regulated were significantly associated with the immune response and signalling pathway. Conversely, the down-regulated target genes of lncRNAs were significantly associated with oxidation-reduction processes and metabolic processes [12] (Table 1).

**Table 1.** Differentially expressed lncRNAs between the old liver and the young liver.

LncRNA (References)	Samples studied	Target genes and function	Differentially expression lncRNAs
NEAT1, MEG3, Rian, Mirg [45]	Balb/C mice (*n* = 3)	Inflammation, cellular proliferation, and metabolism	Up-regulated
ENSMUST00000144661.2, ENSMUST00000180730.3, ENSMUST00000181906.1, LNC_000027, LNC_000204, LNC_000366, LNC_000150, and LNC_000426 [64]	Mice (*n* = 50)	Ces1g, Neu1, Dhtkd1, Card6, Gstm3, Bco2, VLDLR, BLVRB, Cyp2c44, Sort1	Up-regulated/Down-regulated
MITAl [65]	Mice (*n* = 10) and Cell lines (HepG2, A549, U87, PC3, Huh7, HCCLM3, SK-Hep1, SMMC-7721, LO2, HGC27, and U251)	Slug (snail family zinc finger 2) transcription	Up-regulated
MSTRG.177174 [12]	Pig (*n* = 4)	SARDH (oxidation – reduction process)	Up-regulated
MSTRG.85782 [12]	Pig (*n* = 4)	IRG6 and ISG15 (defence response to virus) S1PR3 (regulation of interleukin-1 beta production) IRG6 (negative regulation of viral genome replication)	Up-regulated
MSTRG.58269 [12]	Pig (*n* = 4)	DMBX1 (DNA binding) GUCA1A (calcium sensitive guanylate cyclase activator activity)	Up-regulated
MSTRG.205482 [12]	Pig (*n* = 4)	CYP1A1 (hydrogen peroxide biosynthetic process) LIPK (related to lipid metabolic process) SULT1B1 (related to flavonoid metabolic process) TLR4 (related to lipopolysaccharide receptor activity) ENPP3 (nucleotide diphosphatase activity)	Down-regulated

**Figure 2.**
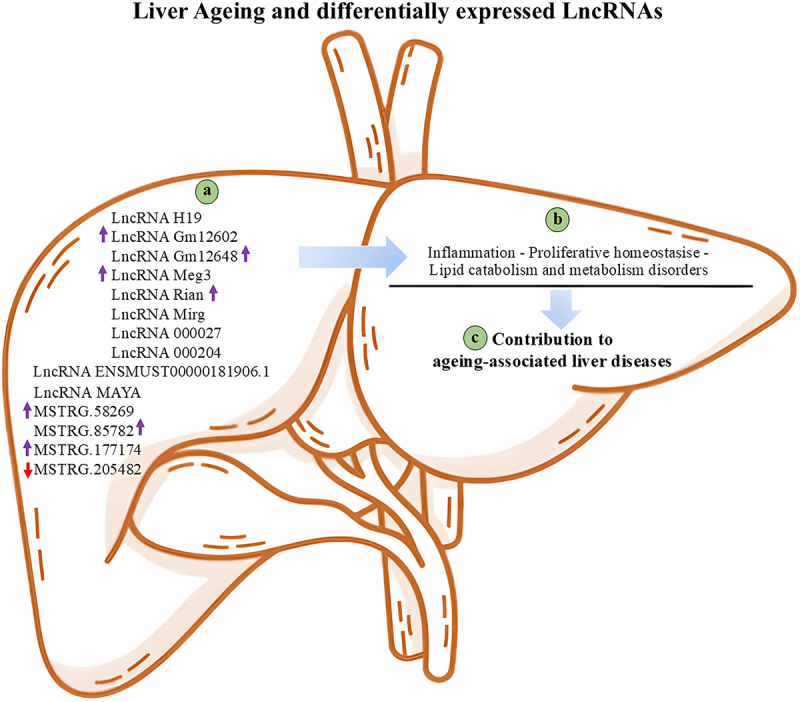
Differentially expressed lncRNAs in ageing-associated liver and their function.

Recent studies have demonstrated that lncRNAs, competitive endogenous RNAs (ceRNAs) with miRNA response elements, can compete with mRNAs by competitively binding to shared miRNAs. Consequently, gene expression is influenced [66]. A variety of lncRNAs modulate gene expression through diverse interactions with DNA, miRNAs, mRNAs, or proteins [67]. Bioinformatics methods have been used to construct a NAFLD-associated lncRNA-miRNA-mRNA network (NLMMN). The objective here is to identify functional lncRNAs that have a role in the progression of NAFLD, since there may be a correlation between lncRNAs and the ceRNA mechanism [66]. A study revealed that lncRNAs primarily influence the extracellular matrix (ECM) via the ceRNA network, thereby impacting the progression of NAFLD. The upregulation of two lncRNAs (LINC00240 and RBMS3-AS3) and the downregulation of one lncRNA (ALG9-IT1) in the findings suggests their potential involvement in the pathogenesis of NAFLD. However, only LINC00240 exhibited substantial upregulation in HepG2, along with lipid accumulation. Consequently, LINC00240, RBMS3-AS3, and ALG9-IT1 May represent new functional lncRNAs that mitigate liver fibrosis in NAFLD by influencing the ECM via the ceRNA network [66]. The progression of chronic liver diseases, irrespective of their underlying causes, is accompanied by the development of liver fibrosis. Abnormal ECM substances, namely collagens and alpha-smooth actin proteins, which are synthesized by liver myofibroblasts, serve as an indicator of fibrogenesis in the liver [68]. In Figure 2, differentially expressed lncRNAs in the liver associated with ageing are displayed.

LncRNA H19 has been shown to be involved in the pathogenesis of ageing liver fibrosis [67]. There is evidence that lncRNA H19 overexpression disrupts intestinal barrier function through miR675, thereby contributing to the progression of NAFLD [69]. Furthermore, lncRNA H19 impairs the functionality of Paneth and goblet cells by inhibiting autophagy [70]. Researchers found that lncRNA Gm2199 was significantly downregulated in injured livers and hepatocytes, and this aberrant downregulation may contribute to impaired hepatocyte proliferation. Overexpression of Gm2199 can restore the diminished proliferation capacity and ERK1/2 expression in damaged hepatocytes [71]. The liver’s ageing process involves a variety of lncRNAs, including Gm12602, Gm12648, Meg3, Rian, and Mirg [72]. In a study conducted in 2021, the role of lncRNAs in prolonged liver ageing was evaluated in an animal model. At first, the lncRNA profiles of 8-month-old SAMP8 and SAMR1 mouse liver genomes were evaluated through deep RNA sequencing. Out of the 2,182 lncRNAs identified, 28 showed differential expression in SAMP8 and SAMR1 mice. LncRNAs that have the potential to participate in liver ageing and related disorders, such as ENSMUST00000144661.2, ENSMUST00000180730.3, ENSMUST00000181906.1, LNC_000027, LNC_000204, LNC_000366, LNC_000150, and LNC_000426, were identified. These lncRNAs exhibited characteristics including reduced full length and open reading frame length, fewer exons, less conservation, and lowered expression levels [64]. These characteristics have been observed in other species, such as humans [73], goats [74], and pigs [75], suggesting the significance of lncRNA in gene regulation, control, and orientation. Analysis using Gene Ontology (GO) and the Kyoto Encyclopedia of Genes and Genomes (KEGG) revealed that these significantly dysregulated lncRNAs were involved in various aspects of liver ageing and ageing-related liver diseases, including retinol metabolism, lipid catabolic and metabolic pathways [64]. Retinol is a derivative of vitamin A and a form of retinoid [76]. Hepatic stellate cells are the repository for approximately 90–95% of retinoids [77]. Changes in the metabolism and release of retinoids by hepatic stellate cells lead to liver damage, which includes hepatic fibrosis and fatty liver [76]. Another study employed a humanized mouse model, which involved repopulating the liver with human hepatocytes, to examine the function of non-conserved human hepatic lncRNAs in NAFLD and metabolic homoeostasis. LINC01018 has been identified as an obesity-related lncRNA, and hLMR1 is demonstrated to enhance the transcription of genes involved in cholesterol biosynthesis [78,79]. The impact of regulatory T (Treg) cells on various ageing-associated liver diseases is intriguing [80]. Altre, a lncRNA present in regulatory T cells, is another significant factor that increases with ageing. Altre regulates mitochondrial dynamics to sustain immune-metabolic homoeostasis in the liver throughout ageing. Consequently, the depletion of Altre results in dysregulated lipid metabolism, increased reactive oxygen species (ROS) accumulation, and an inflammatory liver microenvironment in aged mice [80].

Recent investigations have shown the pivotal function of lncRNAs in the aetiology of liver tumours. Growing evidence confirms that lncRNA H19, an endogenous noncoding single-stranded RNA, acts as an oncogene in the genesis and progression of liver cancer [81]. LncRNA H19 behaves as an oncogene by modulating epigenetic modification, the lncRNA H19/miR-675 axis, the miRNA sponge, drug resistance, and a variety of signalling pathways [82]. Researchers on human liver cancer cell lines found that upregulating the expression of lncRNA H19 and miR-675 May block the Akt/GSK-3/Cdc25A signalling pathway. This could prevent the migration and invasion of these cells in other tissues [83]. Multiple experiments prove that H19 functions as an independent lncRNA that regulates the expression of miR-675, which has an oncogenic effect in hepatocellular carcinoma. It modulates several biological processes via the H19/miR-675 axis by influencing oncogenic or tumour-suppressive factors, since miR-675 has multiple targets and governs different signalling pathways [83]. The H19/miR-675/PPARα axis regulates liver cell damage, energy metabolism, and remodelling caused by hepatitis B X protein. This process occurs through modification of the Akt/mTOR signalling pathway [84]. Further investigation revealed that liver cancer tissues had lower miR-675 expression levels than healthy liver tissues and that overexpressing miR-675 inhibited the proliferation of liver cancer cell lines while promoting apoptosis [85].

The involvement of lncRNAs in the regulation of adenosine monophosphate-activated protein kinase (AMPK) signalling pathways and their association with ageing-related diseases has recently emerged as a significant area of research for scientists. LncRNAs exhibit multiple interaction mechanisms with AMPK. Some lncRNAs change AMPK activity by interacting directly with components of the AMPK complex. Other lncRNAs change AMPK signalling pathways by regulating proteins that are either upstream or downstream of AMPK, directly or indirectly [86].

AMPK plays a crucial role in regulating cellular energy homoeostasis and serves as a primary indicator for nutrients and energy levels [87]. Prior research indicates that AMPK signalling pathways play a role in the regulation of ageing and age-related diseases [88]. Investigations confirm that the potential for AMPK activation and its expression levels decline with ageing [89]. Research utilizing animal models indicates that AMPK phosphorylation levels are markedly lower in ageing mice relative to their younger counterparts [90]. Both rodents and humans experience suppression of AMPK activity, primarily due to excessive caloric intake, insufficient physical activity, increased inflammation, and the effects of ageing [91–97]. Numerous studies demonstrate a significant correlation between decreased AMPK activity and the occurrence of metabolic diseases, such as cardiovascular, obesity, diabetes, and NAFLD [98,99]. The lncRNA metabolism-induced tumour activator-1 (MITA-1) functions as a chromatin-enriching lncRNA. The LKB1-AMPK signalling pathway epigenetically induced MITA-1 in nutrient-deprived conditions. MITA-1 significantly promotes the metastasis of hepatocellular carcinoma by facilitating epithelial – mesenchymal transition (EMT), which is an early and essential process in cancer metastasis. Additionally, MITA-1 expression was found to be up-regulated in hepatocellular carcinoma. The deletion of this factor inhibited the metastasis of the cancer cells both in vitro and in vivo. The findings elucidated the regulation mechanism of lncRNA-mediated AMPK pre-tumorigenic activity, connecting energy stress with cancer spread, and identified a prospective therapeutic target for liver cancer. The mechanism by which MITA-1 facilitated EMT may have been influenced by the increased transcription of snail family zinc finger 2 (Slug). The reduction of MITA-1 resulted in decreased Slug expression, whereas the overexpression of Slug significantly mitigated the impact of MITA-1 deletion on the migration and invasion of liver cancer cells. Consequently, MITA-1 May promote EMT by upregulating Slug transcription, suggesting that the AMPK/MITA-1/EMT pathway could serve as a potential therapeutic target for hepatocellular carcinoma [65].

## 5. Conclusion

LncRNAs play an important role in physiological processes, especially in diseases such as cancer and cellular ageing. The present review attempts to provide valuable insights into the underlying mechanisms of cellular senescence and the development of age-related liver disorders. As it turned out, lncRNAs play an important role in liver regeneration through specific regulatory mechanisms such as MALAT1 and lncRNA-Hand2. In addition, lncRNAs have been found to play a very important role in regulating vital pathways necessary for the repair and growth of liver tissue. The evolving understanding of lncRNAs provides new insights into the molecular processes governing liver health and ageing and paves the way for potential therapeutic interventions targeting these non-coding RNA molecules to alleviate age-related liver dysfunction and disease. Further research on the roles and mechanisms of lncRNAs in liver ageing promises to advance our knowledge of ageing-related liver pathology and strengthen disease management strategies in the elderly population.

## Author contribution statement

Peyman Kheirandish Zarandi: conceptualization, writing-original draft preparation, data curation. Mohsen Ghiasi: writing-original draft preparation, data curation, and figure. Mohammad Heiat: project administration, supervision, conceptualization, writing-reviewing and editing. All authors contributed to the paper and approved the submitted version.
